# Morphological Analysis and Bond Strength to Root Canal **Dentin of Endodontically Treated and Retreated Teeth: An Ex Vivo Study**

**DOI:** 10.3290/j.jad.b5780319

**Published:** 2024-10-14

**Authors:** Mario Alovisi, Pietro Palopoli, Allegra Comba, Leandro Allais, Andrea Roggia, Andrea Baldi, Damiano Pasqualini, Elio Berutti, Nicola Scotti

**Affiliations:** a Dentist, University of Turin, Department of Surgical Sciences, Dental School, Endodontics, Turin, Italy. Conceptualization of the study design, supervised every step of the study, proofread the manuscript.; b Dentist, Politecnico di Torino, Turin, Italy. Conceptualization of the study design, conducted the experimental procedures, wrote the main manuscript, proofread the manuscript.; c Dentist, University of Turin, Department of Surgical Sciences, Dental School, Endodontics, Turin, Italy. Conceptualization of the study design, supervised every step of the study, performed statistical evaluation, proofread the manuscript.; d Dentist, University of Turin, Department of Surgical Sciences, Dental School, Endodontics, Turin, Italy. Conceptualization of the study design, conducted the experimental procedures, proofread the manuscript.; e Dentist, University of Turin, Department of Surgical Sciences, Dental School, Endodontics, Turin, Italy. Conceptualization of the study design, conducted the experimental procedures, proofread the manuscript.; f Dentist, University of Turin, Department of Surgical Sciences, Dental School, Endodontics, Turin, Italy. Conceptualization of the study design, proofread the manuscript.; g Dentist, University of Turin, Department of Surgical Sciences, Dental School, Endodontics, Turin, Italy. Conceptualization of the study design, supervised every step of the study, proofread the manuscript.; h Dentist, University of Turin, Department of Surgical Sciences, Dental School, Endodontics, Turin, Italy. Conceptualization of the study design, supervised every step of the study, proofread the manuscript.; i Dentist, University of Turin, Department of Surgical Sciences, Dental School, Endodontics, Turin, Italy. Conceptualization of the study design, supervised every step of the study, proofread the manuscript.

**Keywords:** bond strength, confocal laser scanning microscopy, radicular dentin, self-etch resin cement, self-adhesive resin cement

## Abstract

**Purpose:**

To assess the bond strength and the hybrid layer (HL) micro-morphological characteristics at the cement-dentin interface (CD-i) between root canal walls and two adhesive resin cements [self-etch (SERc) and self-adhesive (SARc)] in root-canal-treated (RCT) and naturally aged retreated teeth (RCR-T).

**Materials and Methods:**

Vital (n = 16) and RCT (n = 16) teeth were, respectively, endodontically treated or retreated. Fiber posts were luted either with SERc (Clearfil Universal Bond Quick + DC Core Plus) or SARc (iCEM). Samples were then sectioned into 1 mm thick slices perpendicular to the fiber post and submitted to push-out bond strength test. Vital (n = 4) and RCT (n = 4) first maxillary molars were also selected and prepared to evaluate CD-i morphology through confocal laser scanning microscopy (CLSM). Three-way analysis of variance (ANOVA) and Tukey post-hoc tests were assessed to statistically analyze the obtained data (p <0.05).

**Results:**

Bond strength was significantly jeopardized in retreated teeth and in the root apical half, while the cements had no significant influence. Most failures occurred between dentin and resin cement. HL thickness was also hindered in retreated teeth. iCEM produced a thinner HL compared to SERc. Resin tag formation was significantly hampered in the root apical half.

**Conclusions:**

SARc performed as well as SERc on aged RCT radicular dentin. Clinicians can rely on simplified one-step luting systems when adhesion is required in unfavorable substrates such as the root canal post space of aged RCT teeth.

The use of post and core restorations is limited in modern dentistry. However, the rehabilitation of heavily compromised root-canal-treated (RCT) teeth, with no residual coronal structure still appears to benefit from the insertion of a radicular post^3,24^ to enhance core-retention and resistance to flexural stresses. In the past years, metal cast posts and cores have been routinely used.^3,14,24^ However, thanks to the drastic improvement of adhesive technologies over years and the consequent need to reduce clinical invasiveness toward sound structure, metal-free posts increased their popularity.^3,15,45^ Bonding to dentin, a process traditionally achieved through the etching of the substrate followed by the application of a primer and an adhesive resin (etch-and-rinse technique), is recognized as a consolidated acquisition. The resin infiltration of inter-tubular dentin, commonly known as the hybrid layer (HL), and the resin penetration into dentinal tubules (resin tags) are the mechanisms that allow the micro-mechanical resin-dentin interlocking.^17,29^ More recently, self-etch adhesives in association with resin cement (SERc) and self-adhesive resin cements (SARc) have been introduced to simplify the clinical steps required for adhesion. Some studies affirm that these approaches could perform even better than the traditional etch-and-rinse systems in unfavorable cavity configurations such as root canals.^25,30,37,42^ Indeed, RCT dentin is an ideal substrate for adhesive techniques, markedly in its apical portion.^25^ The unfavorable root canal geometric configuration,^43^ the consequential uncontrolled resin polymerization shrinkage,^43^ the effects of endodontic procedures on the dentinal substrate,^28^ the complicated removal of debris produced by post-space preparation^33^ and humidity control are only a few of the challenges to overcome.^25^ Moreover, histological modification of dentin over years, once the pulp tissue is removed, represent an additional issue. Previous studies reported how age-related substrate alterations may significantly affect the bonding ability of adhesive materials.^23,34^ In RCT teeth, the aging histological modifications, as the accumulation of minerals in peri-tubular dentin^27^ and the collagen cross-linking in inter-tubular dentin,^46^ appear to be relatively fast,^46^ suggesting an increased challenge in adhesive procedures. Nonetheless, non-surgical root canal retreatment procedures may further jeopardize radicular dentin bonding potential in root canal retreated teeth (RCR-T).^44^ However, only a few studies assessed the bonding capacity of radicular dentin of RCR-T teeth, and none of them used specimens with a history of natural aging and function.^16,31,35^

Therefore, the purpose of this *in vitro* study was to assess the bond strength and the micro-morphologic characteristics, in terms of HL thickness and number of resin-filled dentinal tubules, of the adhesive interface between root canal dentin and two resin cements (SERc and SARc) in root-canal-treated and naturally aged retreated teeth.

The tested null hypothesis were that: (1) the quality of the substrate (freshly endodontically treated and aged root canal retreated dentin) has no influence, in terms of bond strength and micro-morphology of the adhesive interface, on the adhesion of resin cements; (2) the topography of the substrate (coronal versus apical half of the root canal post space) has no influence, in terms of bond strength and micro-morphology of the adhesive interface, on the adhesion of resin cements; (3) the type of resin cement (SERc vs SARc) has no influence on the bond strength and micro-morphology of the adhesive interface.

## Materials and Methods

The manuscript of this laboratory study has been written according to Preferred Reporting Items for Laboratory studies in Endodontology (PRILE) 2021 guidelines.

### Study Design

The general description of the main materials used in the present study, their manufacturers and composition are listed in Table 1. This study was designed in four study groups, where the specimens were randomly allocated (www.randomizer.org) considering:

**Table 1:** Materials and techniques employed for fiber post cementation

Material	Type	Composition	Post treatment [manufacturer instruction (MI)]	Post space treatment (MI)
Clearfil Universal Bond Quick (C Universal bond) (bond) + Clearfil DC Core Plus (Clearfil DC) (paste) (Kuraray Noritake; Okayama, Japan)	Self-etch (SE)	Bond: Bis-GMA (10–25%), ethanol (10–25%), HEMA (2.5–10%), 10-MDP, hydrophilic amide monomer, colloidal silica, silane coupling agent, sodium fluoride, camphorquinone, water Paste: Bis-GMA, TEGDMA, hydrophobic/hydrophilic aliphatic dimethacrylate, hydrophobic aromatic dimethacrylate, silanated barium glass filler, silanated colloidal silica, colloidal silica, aluminum oxide filler, CQ, accelerator, initiator	Apply phosphoric acid [5 seconds (s)]; rinse and dry; apply bond, then dry by blowing mild air	SE mode: apply bond with a rubbing motion (no waiting time); dry by blowing mild air and paper point until bond does not move; LED light cure (10 s); squeeze paste; insert the post and light cure paste (20 s)
iCEM (Kulzer, Hanau, Germany)	Self-adhesive (SA)	Acidified urethane and di-, tri-, multifunctional acrylate monomers; 41% filler by weight	None	Rinse the post space with water; dry with mild air and paper points, without over-drying; squeeze paste; insert the post and light cure paste (40 s)

1. Status of the specimens: a) extracted vital (VT) and b) and non-vital teeth (RCT) for which the donor had documented clinical evidence confirming they had undergone root canal treatment at least 15 years earlier, were collected to prepare samples for push-out testing and confocal laser microscopy.
2. Adhesive approach performed for fiber post cementation: a) self-etch approach (Clearfil Universal Bond Quick + Clearfil DC Core Plus, Kuraray Noritake; Okayama, Japan); and b) self-adhesive approach (iCEM, Kulzer, Hanau, Germany).

### Sample Preparation

Permanent straight single-rooted vital and RCT human teeth of similar length and anatomy, extracted for periodontal reason in patients aged between 45 and 55, were collected in accordance with the local ethics committee (protocol number CS2/0187). A sample size of 16 per group was calculated with G*Power 3.1.4 (Kiel University, Kiel, Germany) considering α-error = 0.05 and ß = 0.95. For the RCT teeth, specimens were collected only when precise information on the timing of the former endodontic treatment was present, and only teeth endodontically treated at least 15 years earlier were included in the study. Moreover, for the same group, teeth that did not have gutta-percha filling were also excluded from the study. Roots with cracks, resorption, or immature apices were discarded. After debriding the root surface, specimens were stored in 0.1% thymol at 4°C. Roots were sectioned, perpendicularly to the long axis of the tooth, at the level of the cemento-enamel junction (CEJ) with a low-speed diamond saw (Isomet 1000, Buehler, Lake Bluff, IL), to obtain a root length of 15 mm. Teeth with oval-shaped canals were excluded.

For the vital extracted teeth, manual scouting and mechanical glide-path were obtained, respectively, with #8-10 K-Files (Dentsply Sirona, Ballantyne, NC, USA) and ProGlider (Dentsply Sirona) up to full working length (WL). WL was recorded when the file tip became visible at the apical foramen under 10× magnification (Pro Ergo; Carl Zeiss, Oberkochen, Germany). Final shaping was performed with ProTaper Next X1, X2, and X3 (Dentsply Sirona). Throughout the shaping procedures, root canals were irrigated with 10% ethylenediamine tetraacetic acid (EDTA) (Tubuliclean; Ogna, Muggiò, Italy) alternated with 5% sodium hypochlorite (NaOCl) (Niclor 5; Ogna) delivered with a 2-mL syringe and a 22-gauge needle. In RCT specimens, gutta-percha was removed with the aid of a D-limonene and 1,2 dichloropropane-based solvent (GPR; Ogna, Muggiò, Italy), and shaping procedures were carried out as previously reported. Root canals were then dried with sterile paper points and sealed with dedicated gutta-percha points (ProTaper Next conform fit; Dentsply Sirona) and endodontic cement (Pulp Canal Sealer EWT; Kerr, Sybron, Romulus, MI, USA) warm vertically compacted. Specimens were then stored in 100% humidity at 37°C.

Forty-eight hours (h) later 10 mm of coronal gutta-percha were removed with D.T. Light Post Universal and Finishing Drill #1 (VDW, Munich, Germany). Post spaces were thoroughly cleaned with 5 mL of distilled water delivered with a 22-gauge needle. Tapered fiber posts (D.T. Light Post #1; VDW) were tried inside the canal to ensure they could reach the desired length without binding to root canal walls. RCT and RCR-T specimens were then randomly allocated in two groups according to the adhesive protocol used to cement the fiber posts (Table 1). Cements were light-cured 60 s after insertion to allow their chemical setting. Light curing was performed using a LED lamp (Valo, Ultradent, USA) leaned against the post-head to standardize its distance from the root canal. After cementation the samples were stored for 24 h in 100% humidity at 37°C.

### Push-out Bond-Strength Analysis and Failure Mode Evaluation

Each specimen was then sectioned perpendicularly to its long axis with a 0.35 mm diamond saw (Micromet; Remet, Bologna, Italy) at slow speed with water cooling to obtain 6 slices (3 coronal and 3 apical), generating eight subgroups (Table 2).

**Table 2:** Specimen sorted by type of endodontic treatment, luting cement, and area of the post space

Treatment	Cement	Area	Subgroups
RCT (n = 16)	iCEM (n = 8)	coronal (n = 24)	TIC
		apical (n = 24)	TIA
	Clearfil DC (n = 8)	coronal (n = 24)	TDC
		apical (n = 24)	TDA
RCR-T (n = 16)	iCEM (n = 8)	coronal (n = 24)	RIC
		apical (n = 24)	RIA
	Clearfil DC (n = 8)	coronal (n = 24)	RDC
		apical (n = 24)	RDA

TIC (treatment-iCEM-coronal) group; (B) (treatment-iCEM-apical) group; TDC (treatment-Clearfil DC-coronal) group; TDA (treatment-Clearfil DC-apical) group; RIC group (retreatment-iCEM-coronal); RIA (retreatment-iCEM-apical) group; RDC (retreatment-Clearfil DC-coronal) group; RDA (retreatment-Clearfil DC-apical) group.

The push-out test was performed by applying an axial load (apical to coronal) to the post at a crosshead speed of 0.5 mm min-1 using an Instron Machine I (model 10/D; Sintech, MTS, Canton, MA, USA). The maximum failure load was recorded in Newtons (N). Push-out bond strength was calculated in megapascal (MPa) by dividing the failure load (N) by the area of the bonded interface (SL) estimated from the formula for calculating the lateral surface area of a truncated cone: SL = π (R + r) [h^2^ + (R − r)^2^]0.5, where π represents the constant (3.14), R is the coronal post radius, r is the apical post radius and h is the slice thickness. The latter was measured using a digital caliper, while the radii were calculated with ImageJ 1.35 S software (National Institutes of Health, Bethesda, MD, USA) from photographs taken with a stereomicroscope (Discovery V 12, Carl Zeiss).

After the push-out test, all samples were analyzed with the stereomicroscope at 40× magnification by a single trained operator, to assess the type of failure. Failures were classified as follows: A, adhesive failure between dentin and resin cement; C, cohesive failure within resin cement, and M, mixed failures including cement and the dentin-cement interface. As cohesive failures within the post or within the dentin did not occur, they were not included in the classification. Within each group, failures were expressed as percentages.

### Micro-morphologic Analysis of the Adhesive Interface Through CLSM Analysis

Four permanent vital and four RCT human first maxillary molars were selected as previously described. Specimens were endodontically treated and retreated as shown before. The same materials and procedures were employed to cement fiber posts. Specifically, fiber posts were luted with Clearfil DC in the palatal canal and with iCEM in the disto-buccal canal of each specimen. Before insertion of the posts, the adhesive system (C Universal Bond) was labeled with 0.1% fluorescein (FNa; Sigma Aldrich, Steinheim, Germany) and the resin cements (Clearfil DC and iCEM) were labeled with 0.1% rhodamine isothiocyanate (RITC; Sigma Aldrich, St Louis, MO, USA). Both the dyes were added by means of a simple mixing process in a ratio of 0.1%.2 To perform the analysis, the roots were perpendicularly sectioned, below the CEJ into slices of less than 1 mm thickness. The slices were then sorted in coronal and apical to obtain 6 slices (3 coronal and 3 apical), generating the same eight subgroups as seen in Table 2. The slices were then positioned, with the coronal side upward, on a microscope slide for grinding and polishing. To this purpose, a series of silicon carbide abrasive papers (1200, 2400, 4000 grit) using running tap water as a lubricant, have been employed. The samples were kept humid during the whole study.

Confocal laser scanning microscopy (CLSM) imaging was performed using a Leica SP8 confocal system (Leica Microsystems) equipped with an argon ion and a 561 nm diode-pumped solid-state (DPSS) laser. Samples were imaged using a HCX PL APO 40×/1.25 NA oil immersion objective. Series of x-y-z images (typically 0.145*0.145*1 µm^3^ voxel size) were collected. Laser power and detector gain were set on the control sample and kept the same for all conditions of the experiment. Images were recorded at a random area of each sample.

Images were analyzed with ImageJ 1.35 S software. Specifically, the thickness of the HL was recorded at four randomly chosen locations and a mean value was obtained. The number of dentinal tubules penetrated by resin cements and adhesive was counted.

### Statistical Analysis

After ascertaining the normality (Shapiro–Wilk test) and homoscedastic (modified Levene test) assumptions of the data sets, the radicular bond strength data were analyzed with a three-way analysis of variance to examine the effects of the age of the endodontic treatment (new treatment/retreatment), cement employed and root area and the interaction of those three factors on micro push-out bond strength. Post-hoc pairwise comparisons were performed using the Tukey test. Chi-square test was used to analyze differences in the failure modes.

Evaluation of the data obtained from confocal microscope was performed comparing the samples of the different groups and subgroups with respect to the morphologic characteristics (hybrid layer thickness, number of tags penetrated with adhesive and resin cement) and calculating the mean values for each slice followed by statistical analysis using the three-way analysis of variance (ANOVA) test and Tukey post-hoc analyses.

For all tests, statistical significance was pre-set at α = 0.05. All statistical analyses were performed using Stata 12.0 (StataCorp, College Station, Texas, USA).

## Results

### Push-out Test

Bond strength data were expressed as means and standard deviations and summarized in Table 3. Results of the three-way ANOVA showed a significant difference for the variable “endodontic treatment” (RCT/RCR-T) (p = 0.01) and “root area” (apical/coronal) (p = 0.003) as well as for the interaction between the cement employed and the root area (p = 0.023). The factor “cement” had no effect on the push-out bond strength (p > 0.05). Tukey post-hoc test showed that bond strength is significantly higher in freshly endodontically treated teeth compared to retreated teeth, independently from the cement employed and the root area considered. In addition, root coronal dentin showed bond strength values significantly higher than root apical dentin.

**Table 3:** Bond strength values (expressed in MPa) according to different groups. Values are expressed as mean (± standard deviation)

	iCEM RCT	Clearfil DC RCT	iCEM RCR-T	Clearfil DC RCR-T
C	12.35 (± 2.49)^a,1^	10.85 (± 3.07)^ab,1^	10.53 (± 3.33)^ab,1^	9.33 (± 3.43)^b,1^
A	9.86 (± 3.18)^a,2^	9.70 (± 2.4)^a,1^	8.48 (± 2.37)^a,1^	9.88 (± 1.61)^a,1^

C and A stand, respectively, for coronal and apical. Within each line, different superscript letters indicate statistically significant differences. Within each column different superscript numbers indicate statistically significant differences (p < 0.05)

### Failure Mode

Failure modes’ distribution of the debonded specimens, expressed as percentages of the total number of specimens tested, are summarized in Table 4. Statistical analyses showed a predominance of adhesive failures between dentine and resin cement in all groups (p <0.05), followed by mixed failures.

**Table 4:** Failure modes after push-out test. Values are expressed as percentage (%)

	TIC	TIA	TDC	TDA	RIC	RIA	RDC	RDA
Adhesive	75	14.28	66.67	28	68	57.14	86.20	47.62
Cohesive	10.71	35.71	20	44	20	23.8	10.34	23.80
Mixed	14.28	50	6.6	28	12	19.04	3.44	28.57

TIC (treatment-iCEM-coronal), TIA (treatment-iCEM-apical), TDC (treatment-Clearfil DC-coronal), TDA (treatment-Clearfil DC-apical), RIC (retreatment-iCEM-coronal), RIA (retreatment-iCEM-apical), RDC (retreatment-Clearfil DC-coronal), RDA (retreatment-Clearfil DC-apical)

### CLSM Analysis

The hybrid layer thickness was significantly influenced by the factors “endodontic treatment” (RCT/RCR-T) (p = 0.03) and “resin cement” (Clearfil DC/iCEM) (p = 0.001). The factor “root area” had no effect on the thickness of the hybrid layer (p > 0.05).

**Fig 1:**
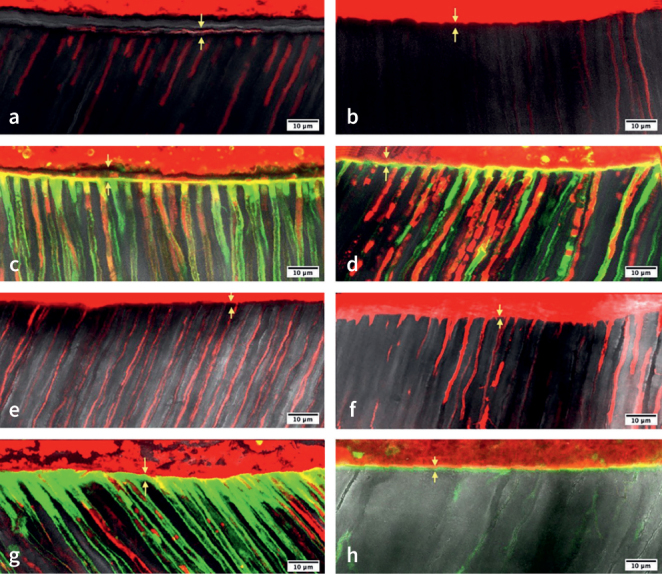
Representative images of HL thickness for the different groups. *(a)* TIC (treatment-iCEM-coronal) group; *(b)* TIA (treatment-iCEM-apical) group; *(c)* TDC (treatment-Clearfil DC-coronal) group; *(d)* TDA (treatment-Clearfil DC-apical) group; *(e)* RIC group (retreatment-iCEM-coronal); *(f)* RIA (retreatment-iCEM-apical) group; *(g)* RDC (retreatment-Clearfil DC-coronal) group; *(h)* RDA (retreatment-Clearfil DC-apical) group.

The hybrid layer thickness of freshly devitalized teeth proved to be significantly higher than that of retreated teeth. Further, post-hoc test showed that iCEM cement produced a hybrid layer thinner than that of Clearfil DC (Table 5). Representative samples of HL formation and filled tubules are shown in Figure 1.

**Table 5:** HL thickness (expressed in µm) according to different groups. Values are expressed as mean (± standard deviation)

	iCEM RCT	Clearfil DC RCT	iCEM RCR-T	Clearfil DC RCR-T
C	2.04 (±0.98)^a,1^	3.45 (±1.20)^a,1^	1.27 (±0.79)^b,1^	3.27 (±1.21)^a,1^
A	1.72 (±1.26)^ab,1^	3.34 (±1.48)^a,1^	0.94 (±0.56)^b,1^	2.55 (±1.70)^ac,1^

C and A stand, respectively, for coronal and apical. Within each line, different superscript letters indicate statistically significant difference. Within each column, different superscript numbers indicate statistically significant difference (p <0.05)

**Fig 2:**
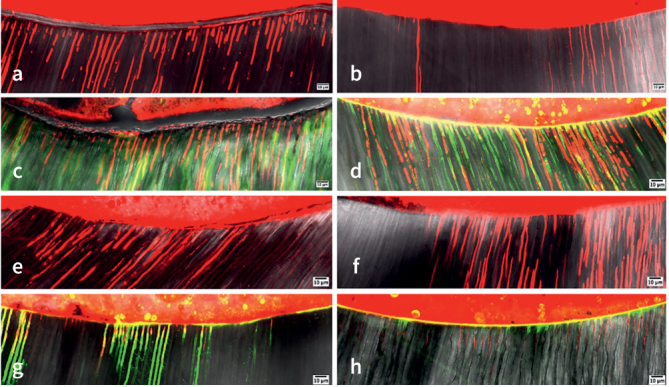
Representative CLSM images of resin cement-infiltrated dentinal tubules. *(a)* TIC (treatment-iCEM-coronal) group; *(b)* TIA (treatment-iCEM-apical) group; *(c)* TDC (treatment-Clearfil DC-coronal) group; *(d)* TDA (treatment-Clearfil DC-apical) group; *(e)* RIC group (retreatment-iCEM-coronal); *(f)* RIA (retreatment-iCEM-apical) group; *(g)* RDC (retreatment-Clearfil DC-coronal) group; *(h)* RDA (retreatment-Clearfil DC-apical) group.

The number of dentinal tubules penetrated with resin cement and adhesive was significantly affected by the root area considered. Coronal slices showed a significantly higher number of penetrated dentinal tubules than apical portion (p < 0.05) (Table 6). Representative images of filled tubules are shown in Figure 2.

**Table 6:** Number of tubules penetrated by adhesive and resin cement. Values are expressed as mean (± standard deviation)

	iCEM RCT	Clearfil DC RCT	iCEM RCR-T	Clearfil DC RCR-T
C	64.76 (±16.45)^a,1^	61.05 (±18.1)^a,1^	62.66 (±16.06)^a,1^	64.6 (±12)^a,1^
A	48.27 (±27.46)^a,1^	64.07 (±12)^a,1^	52.47 (±22.79)^a,1^	50.62 (±20.43)^a,1^

C and A stand, respectively, for coronal and apical. Within each line, different superscript letters indicate statistically significant difference. Within each column, different superscript numbers indicate statistically significant difference (p <0.05)

## Discussion

The challenge of effective bonding to root canal dentin is well described.25 Seemingly, the micro-morphology of the radicular resin-dentin interface (R-DI) and its bond strength have been already investigated in RCT teeth^.2,4,9,32^ Nonetheless, a gap in the literature exists regarding RCR-T teeth, where no evaluations of the R-DI micro-morphology have been performed. Furthermore, few studies investigating the bond strength in RCR-T teeth were conducted by treating and retreating vital specimens,^16,31,35^ and one of them was even carried out on bovine teeth.^16^ However, this procedure implicates that the contribution of natural aging is excluded from the factors that may affect this specific substrate and there is no correspondence with a clinical scenario. Otherwise, in the RCR-T specimens collected in this study for bond strength and R-DI morphologic analysis, the former endodontic treatment was executed at least 15 years earlier, simulating a clinical environment.

Based on the present results, the first null hypothesis should be substantially rejected since the bond strength of freshly endodontically treated dentin was significantly higher than that obtained in naturally aged and devitalized, at least 15 years earlier, teeth undergoing endodontic retreatment (p = 0.01). This is in agreement with the literature,^16,31,35^ even though Pelegrine et al showed that only SERc was affected by the substrate, markedly in the apical portion, and no significant difference was registered for the SARc.31 It is generally accepted that debris and smear layer produced by RCR-T procedures may act as an obstacle between resins and dentinal surface, being difficult to eradicate from the post space.^16^ Guedes et al demonstrated that the solvents used to remove gutta-percha might be detrimental to the adhesive procedures,^16^ since they could penetrate dentinal structure enough to withstand the debriding action of the post space preparation. The softened gutta-percha itself could easily be compacted into dentinal tubules, where it cannot be removed, thus hampering the bond strength of resin cements.^19^ Moreover, it has been reported that gutta-percha solvents can alter the chemical structure (organic and inorganic composition) of dentin, and that any changes in these components can affect the adhesion of restorative materials.^41^ In such regard, it can be expected that the solvent used in the present study may have contributed to the worse bonding performance of the retreated specimens.

As stated before, aged dentin is a less favorable substrate for adhesion^23,34^ and root canal treatment further accelerate the aging process.^46^ One of the main and most evident structural modifications that takes place with aging is the gradual reduction of tubules lumen, due to the accumulation of minerals in peri-tubular dentin.^27^ After an adequate number of lumens have been filled, the tissue appears transparent, as the amount of light scatter off of the lumens decreases, and dentin that has undergone this change is known as “sclerotic.”^21^ Besides the increased amount of mineral content, another important change is represented by the size of the mineral crystallites. They are smaller in transparent dentin than in normal dentin.^21^ Thus, because of the nature of the deposited material filling the lumens, dentin aging leads to an increase in mineral content.^36^ In general, older teeth have a higher mineral-to-collagen ratio compared to young ones and this also accounts for an increased hardness.^27^ Within the inter-tubular matrix, the most important modifications take place in the collagenous microstructure. Type I collagen, once synthesized, undergoes extensive modifications, resulting in a characteristic pattern of cross-links.^40^ These changes in the collagen matrix may increase dentinal fragility, contributing to the structural response.^46^

Therefore, it can be speculated that the aforementioned age-related organic and inorganic structural changes may render the interaction of dentinal substrate with adhesive resins less favorable.

In the present study, the luting cement employed had a significative influence on bond strength only when considering the coronal half of the post space, where SARc was superior to SERc irrespective of the canal treatment (p = 0.023). Pelegrine et al reported similar results, but only in the apical third of the retreated specimens.^31^ In SARc, the methacrylate monomers modified by carboxylic or phosphoric acid groups can condition the dentinal substrate without any etching or bonding pre-treatment. These systems do not require smear layer removal, that is rather modified and infiltrated.^10^ Besides the micro-mechanical interlocking, SARc also interact with calcium ions creating a chemical bond.^10^ Moreover, they are less sensitive to humidity, which may be relevant in areas where moisture control is difficult, such as the post space.^25^ These properties may explain the positive bond strength performance of SARc. Nonetheless, other reports showed different results. Pereira et al discouraged the use of SARc in retreated teeth even though the difference with SERc was not significant.^35^ The heterogeneity of outcomes may be explained, besides the differences in methodologies, by the vastity of luting cements available on the market.

Studies testing different materials often require comparisons. The heterogeneous group of cements available on the market differs in terms of composition, delivery system, setting reaction, setting time, and pH.^26,39^ The bond strength of the SARc used in this study (iCEM) had never been tested in radicular dentin before.

The lack of agreement persists when analyzing the impact of the root area on bond strength. Data obtained in the present investigation reflect the trend according to which adhesion is less effective in the apical portion of the post space (p = 0.003).^16,25,33^ This may be coherent with the increased difficulty to remove debris from the deep area of the root canal, and the decreased number of dentinal tubules available for resin infiltration in this region.^25^ However, these drawbacks may be compensated by the better match between the canal and the post diameter in the apical area. Indeed, the fiber post retentive strength is the result of chemical bonds, micro-mechanical interlocking and sliding friction.^13^ Moreover, the better polymerization of resin cements in the coronal third, due to the proximity of the curing light, is counterbalanced by the higher polymerization shrinkage.^38^ Coherently, other studies reported the highest bond-strength values in the apical portion,^31,47^ while in other reports the root region had no significant influence.^15,31^ A limitation of the present study is that the post space was divided in only two halves: coronal and apical. This has been done in order to simplify procedures.

The failures analysis, assessed after the push-out test, revealed how most fractures were detected between the dentinal surface and the luting cement, namely adhesive failures. These findings are confirmed by other authors.^5,10,16,35^ Guedes et al further investigated adhesive failures through confocal microscopy and reported how the separation occurred between the HL and the resin cement.^15^ The present study does not add data in this context since the CLSM analysis of failures was not a target of the study.

CLSM was rather used to investigate the micro-morphology of the R-DI. As stated by Bitter et al, CLSM is advantageous for the visualization of more detailed information with respect to both penetration and distribution of resin cement and adhesive.^5^ Moreover, in the same study, confocal microscopy, comparing to scanning electron microscopy (SEM), provided comparable results in terms of HL thickness.^5^ In the present investigation, fluorescein and rhodamine were used as dyes as they are easy to distinguish and do not diffuse one into the other.5 However, drawbacks of CLSM are the difficulty of standardizing the dye powder incorporation into the resin and the fact that the dye does not form any covalent bond with the resin. This may lead, respectively, to a not uniform dye distribution and to dye leaching into the hydrophilic dentinal tissue.^7^ As CLSM images of the present study showed homogeneous fluorescence, a uniform distribution of the dye could be assumed. An additional issue is represented by the possible negative effect of the dyes on polymerization and adhesive strength.^7^ However, this does not represent a limitation for the present study as the bond strength analysis was performed on different samples.

CLSM showed that the RCR-T dentinal substrate, compared with the RCT one, produced a significantly less thick HL (p = 0.03) regardless of the luting cement it interacts with. As stated before, there are no studies evaluating the R-DI morphology in retreated specimens to compare these data with. However, these results seem coherent with the abovementioned effect that natural aging and endodontic treatment have on dentin.^16,27,46^ HL thickness was also influenced by the type of luting cement employed. SERc, compared to SARc, produced a significantly thicker HL, regardless of the canal treatment (p = 0.001). This is in line with the literature. As a matter of fact, SARc only interact superficially and do not produce a considerable HL.^1,4,6,11^ It must be pointed out that, due to this only superficial morphological interaction, the HL detection and measurement in CLSM images is challenging when evaluating SARc and its reliability and reproducibility could be questioned. Bitter et al detected hybridization of dentin only sporadically.^6^ Yet, it is important to have a visualization of the micro-morphologic characteristics of the dentin-adhesive interface, and CLSM imaging has proven to provide a reliable estimation of HL thickness.^5^

Conversely, the root topography had no significant influence on the HL morphology, even though, as expected, a tendency of higher values in the coronal region was registered (p > 0.05). These results are supported by other authors.^4,6^

In this study, the aged endodontically retreated substrate did not significantly affect resin tag formation. It may be speculated that the age- and treatment-related changes in inter-tubular dentin interfere more than the tubular characteristics in terms of resin infiltration.^17^ In all groups, resin tags were consistently represented and produced in comparable numbers by the luting cements tested. However, the ability of SARc to penetrate dentinal tubules is questioned by literature. Pelegrine et al, in a SEM investigation, stated that the tested SARc (RelyX U200, 3M ESPE, Seefeld, Germany) did not show any resin tag.31. Similar data were also obtained in a CLSM analysis in which the same luting cement was tested.^6^ Therefore, such deep disagreement with the results of the present study may be mainly attributed to the different type of SARc used (iCEM). Resin tag formation was significantly influenced by the root region considered. It has been thoroughly demonstrated how the number of dentinal tubules decreases toward the apex.^12^ Coherently, significantly less resin-filled tubules are found in the apical portion of a post space.^4,31^ The present study did not deviate from this assumption.

A final consideration is reserved for the irrigation protocol used in the present study. In general, endodontic irrigants do have an impact on the chemo-mechanical properties of dentin and on its bonding potential.^8,18,20,22^ As a matter of fact, NaOCl irrigation may lower the resin-dentin bond strength values of RCT dentin.^28^ When NaOCl is used for root canal irrigation, there might be some reactive free radicals which can cause the incomplete polymerization of monomers.^22^ On the other hand, the possible adverse effects of EDTA on adhesion seem to depend on the bonding system used.^18^ The removal of the smear layer is a disadvantage for adhesion when SERc systems are used.^18^ This has to be taken into account when investigating adhesion on RCT teeth. Therefore, as the use of calcium-chelating agents is an important step in Endodontics, excessive demineralization caused by endodontic irrigation should be avoided when SERc are to be used.^18^ In the present study, a relatively short application of NaOCl and EDTA was performed, simulating endodontic irrigation in an ordinary clinic. Yet, resin tags and HL formation may have also been influenced by the irrigation protocol employed since the demineralization and the deproteinization facilitate the penetration of the resin tags into the dentinal tubules.^18^

A limitation of the study is that, for the RCR-T group, it was not possible to gather precise information on the former endodontic treatments performed at least 15 years earlier. These data, such as the type of sealer used or irrigation protocol, are important as these factors could affect the structure and properties of root dentin.

## Conclusion

Within the limitation of the present study, it can be concluded that resin cement bond strength potential is significantly hampered in an aged root-canal-treated substrate. However, the same substrate did not significantly interfere with hybridization, as resin tags formation was not affected by dentin condition.

Moreover, the present study demonstrated how iCEM SARc showed similar bond strength values compared to SERc.

### Clinical Relevance

The use of simplified single-step luting systems, such as SARc, may be a reasonable option when adhesion is required in anatomical constraints with a heavily modified substrate, such as the post space of RCT-aged teeth. However, further studies are needed to assess the long-term performances of these cements.

## Data Availability

All data and materials can be requested from the corresponding author: nicola.scotti@unito.it.
